# Which patients bring the most costs for hospital? A study on the cost determinants among COVID-19 patients in Iran

**DOI:** 10.1186/s12962-022-00386-9

**Published:** 2022-09-24

**Authors:** Vajihe Ramezani-Doroh, Leili Tapak, Yadollah Hamidi, Saeid Bashirian, Ali Reza Soltanian, Mahyar Motaghed, Ebrahim Ghelichkhani, Elaheh Talebi-Ghane

**Affiliations:** 1 Department of Health Management and Economics, School of Public Health, Hamadan University of Medical Sciences, Hamadan, Iran; 2 Modeling of Noncommunicable Diseases Research Center, Hamadan University of Medical Sciences, PO Box: 65176-19657, Hamadan, Iran; 3 Department of Biostatistics, Hamadan University of Medical Sciences, Hamadan, Iran; 4 Social Determinants of Health Research Center, Hamadan University of Medical Sciences, Hamadan, Iran; 5 Farshchian Hospital, Hamadan University of Medical Sciences, Hamadan, Iran

**Keywords:** COVID-19, Cost, Quantile regression, Iran

## Abstract

**Background:**

Accurate information on the cost determinants in the COVID-19 patients could provide policymakers a valuable planning tool for dealing with the future COVID-19 crises especially in the health systems with limited resources.

**Objectives:**

This study aimed to determine the factors affecting direct medical cost of COVID-19 patients in Hamadan, the west of Iran.

**Methods:**

This study considered 909 confirmed COVID-19 patients with positive real-time reverse-transcriptase polymerase-chain-reaction test which were hospitalized from 1 March to 31 January 2021 in Farshchian (Sina) hospital in Hamadan, Iran. A checklist was utilized to assess the relationship of demographic characteristics, clinical presentation, medical laboratory findings and the length of hospitalization to the direct hospitalization costs in two groups of patients (patients with hospitalization ≤ 9 days and > 9 days). Statistical analysis was performed using chi-square, median test and multivariable quantile regression model at 0.05 significance levels with Stata 14 software program.

**Results:**

The median cost of hospitalization in patients was totally 134.48 dollars (Range: 19.19–2397.54) and respectively 95.87 (Range: 19.19–856.63) and 507.30 dollars (Range: 68.94–2397.54) in patients with hospitalization ≤ 9 days and > 9 days. The adjusted estimates presented that in patients with 9 or less hospitalization days history of cardiovascular disease, wheezing pulmonary lung, SPO2 lower than 90%, positive CRP, LDH higher than 942 U/L, NA lower than 136 mEq/L, lymphosite lower than 20% and patients with ICU experience had significantly positive relationship to the median of cost. Moreover, in patients with more than 9 hospitalization days, history of cardiovascular disease and ICU experience was statistically positive association and age older than 60 years and WBC lower than 4.5 mg/dL had statistically negative relationship to the median of hospitalization cost.

**Conclusion:**

As the length of hospital stay, which can be associated with the severity of the disease, increases, health systems become more vulnerable in terms of resource utilization, which in turn can challenge their responsiveness and readiness to meet the specialized treatment needs of individuals.

## Introduction

Emergency and rapid spread of COVID-19 has putted a huge burden on the individuals’ daily life globally. Imposed limitation on the human's life and the economic hardship in countries [1] from one hand and the less seen increased demand to specialized treatment facilities in the COVID-19 patients on the other hand has faced countries with many difficulties in coping up with this emergency. The studies show a huge economic burden resulted from COVID-19 over all countries [1]. Especially, health systems around the world have experienced many crises and problems in the management of the epidemic during the outbreak of the COVID-19 pandemic. Need to hospitalization for the majority of the infected patients has putted significant burden on the health systems and has caused that the hospitalization costs become a prominent part of disease economic burden [2]. It seems higher hospital admission rate, prolonged length of stay, and need to the specialized treatments [2–4] are among the most probable reasons for increased hospitalization cost among COVID-19 patients. A study in Hamadan city, Iran showed that the median lengths of hospitalization in COVID-19 patients was five days and some demographic and clinical features were related with a higher length of stay in patients [5]. Another study revealed that being more than 60 year old, respiratory rate more than 30 per minute, having comorbidity, and having a prolonged hospitalization, the risk of mortality in COVID-19 Increased [6]. Disease severity and prolonged hospitalization could be related with higher cost in patients. Studies have shown that the hospitalization costs in the COVID-19 patients are different based on different characteristics across countries [2, 7, 8]. Planning for and forecasting the future needs to the financial support especially for the fragile population could empower countries in the management and control of the future crisis caused by the next wave of COVID-19 pandemic [9, 10]. Particularly by considering probable variants of SARS-COV virus and the low rate of COVID-19 vaccine tendency around the world notably in the developing countries [11] there are two sources of concerns regarding possible future waves and the management of COVID-19. Costs features of COVID-19 patients could be used as a tool for better resource allocation. Accurate information on the cost of the infected individuals could provide the policymakers and the managers with insight into the actual used resources and the most driving factors of it [9, 10]. In addition, costs could be used as a valuable planning tool for the financing strategy of health system and designing appropriate payment policies. Understanding the most important cost components of the COVID-19 pandemic and analysis of its determinants could enable targeted policy making in combating the next crisis. There are few studies examined the cost composition of COVID-19 patients and its determinants especially in Iran [2, 8, 12]. So the present study aimed to investigate driving factors of direct hospitalization cost of COVID-19 patients in Hamadan city. The results of this study could be used as a valuable tool in the planning and management of Iranian health system resources in dealing with the future COVID-19 crises.

## Materials and methods

This retrospective observational study considered 909 hospitalized COVID-19 patients who had positive real-time reverse-transcriptase polymerase-chain-reaction test (RT-PCR) on samples which collected from upper respiratory nasopharyngeal swabs. These patients were hospitalized from 1 March to 31 January 2021 in Farshchian (Sina) hospital in Hamadan, a city in the west of Iran. The protocol of this study was approved by Research Council of Hamadan University of Medical Sciences.

The data was gathered from patients’ medical records and their registered payment bills in the hospital by a predetermined checklist. This checklist was contained demographic characteristics, clinical presentation, medical laboratory findings, length of hospitalization and their direct medical costs. Demographic characteristics included gender, age at the diagnosis (older or younger than 60 years), marriage status (married, single, divorced/dead spouse), residency (rural, city), consumption of opiate; clinical presentation included comorbidity disease (no, diabetes, cardiovascular, hypertension, two or more diseases), blood pressure (lower or higher than 90 mm Hg), breath pattern (normal, takipeneh, dispeneh/distress), pulmonary lung [normal, abnormal (Crackling/Rales/Wheezing)], number of breathing (lower or higher than 30 per minute), heart rate (lower or higher than 125 per minute) and electrocardiogram (ECG: normal, abnormal); medical laboratory findings included Oxygen saturation (SPO2: (lower or higher than 90%), C-reactive protein (CRP: negative, positive), Erythrocyte Sedimentation Rate (ESR: normal, abnormal), Creatinine (lower 0.8, 0.8–1.3, higher than 1.3 mg/dL), blood urea nitrogen (BUN: 5–20, higher than 20 mg/dL), partial thromboplastin time (PTT: 25–35, higher than 35 s), platelets count (130–400 × 1000 µL, lower 130 × 1000 µL), prothrombin time (PT: 11–13 s, higher than 13 s), the counts of white blood cells (WBC: 4.5–11 × 1000 µL, lower than 4.5 × 1000 µL, higher than 11 × 1000 µL), neutrophils (40–60%, lower than 40%, higher than 60%), lymphocytes (20–40%, lower than 20%, higher than 40%), monocytes (2–8%, lower than 2%), Hematocrit (HCT with normal Range: 37–47% for women and 42–52% for men), hemoglobin (Hb with normal Range: 12–16 g/dL for women and 14–18 g/dL for men), Lactate dehydrogenase [LDH: lower or higher than 2ULN (942 U/L)], Creatine phosphokinase (CPK: lower or higher than 342 IU/L), glutamic-pyruvic transaminase (SGPT: lower or higher than 37 U/L), SGOT (lower or higher than 45 U/L), Alkaline Phosphatase (ALP: lower or higher than 270 U/L), Potassium Blood Test (K: 3.5–5.1 mEq/L, lower than 3.5 mEq/L, higher than 5.1 mEq/L), Sodium Blood Test (NA: 136–145 mEq/L, lower than 136 mEq/L), Blood sugar (BS, 70–105 mg/dL, higher than 105 mg/dL), hospitalized in intensive care unit (ICU), the length of hospitalization (lower than 9 days and higher than 9 days).

The direct medical costs were gathered by registered payment bills through the hospital information system (HIS). These costs were classified into seven categories as follows: the costs of imaging [computed tomography (CT) scan and Radiography], hoteling (ordinary or intensive bed services), nursing services, physician visits, drugs and supplies, laboratory and finally other services. The outcome in this study was sum of all the hospitalization costs based on US dollars (1 US = 250,000 Rial). It was aforementioned that the costs converted to dollars based on the exchange rate of Rial to Dollar at the study time.

**Fig. 1:**
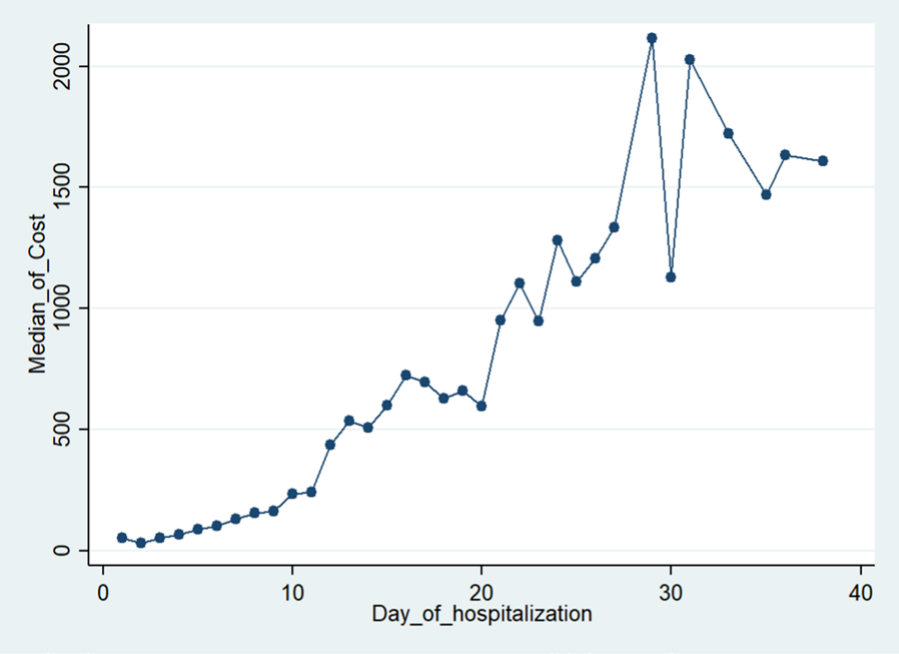
The changes in the median cost of hospitalization based on the length of hospitalization

As previous expectation, the hospitalization cost increased by extending in the hospitalization days. Based on Fig. 1, it was observed that the median of hospitalization cost was started to increase remarkably after the ninth day. Hence, all analyses were performed separately for the patients with hospitalization ≤ 9 days and > 9 days. The characteristics of the patients are described based on these two groups by frequency (%) and compared by chi-square test. The median and interquartile range (IQR) of hospitalization cost in COVID-19 patients were reported and compared in two groups by median test. In the following, unadjusted quantile regression models were applied to estimate the effect of covariates on the median of hospitalization cost in the patients with ≤ 9 and > 9 hospitalization days, separately. Then, we selected variables with P value less than 0.3 to include in the multiple quantile regression model. After fitting multiple quantile regression model, the variables with P value more than 0.3 that their elimination leads to better fitting, were removed from the model. All analyses were performed at 0.05 significance levels using Stata 14 (Stata Corp LP, College Station, TX, USA) statistical software.

## Results

**Fig. 2:**
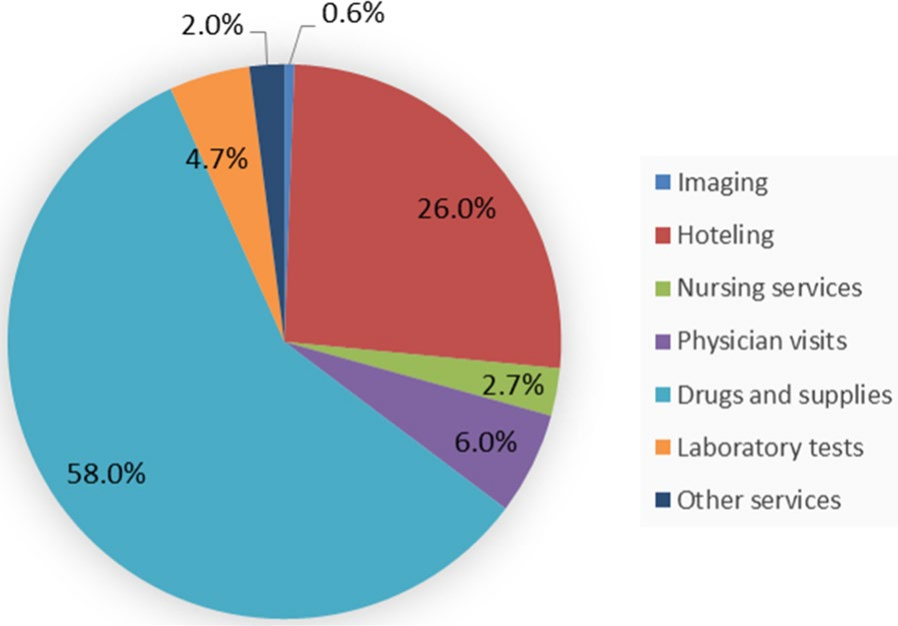
The distribution of various cost categories in the COVID-19 hospitalized patients

The median cost of hospitalization in all COVID -19 patients was 134.48 dollar with range of 19.19–2397.54. The distribution of hospitalization cost is shown in Fig. 2. It is observed that the highest and the lowest%age of cost was spent on the drugs and supplies (58%) and the imaging (0.6%) with the median of 162.47 and 1.14 dollars, respectively.

**Fig. 3:**
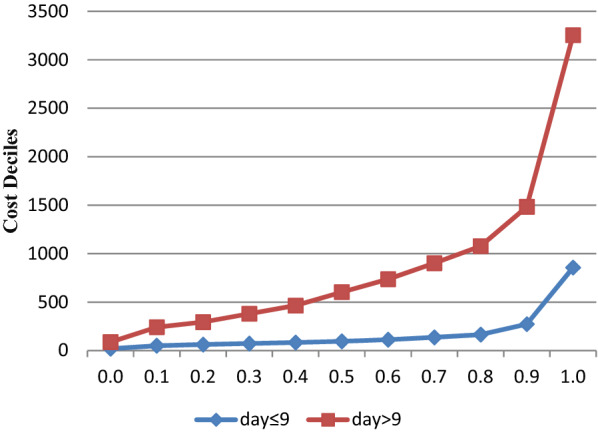
The deciles of costs in the COVID-19 patients with ≤ 9 and > 9 hospitalization days

From 909 patients, 652 (71.73%) patients were stayed 9 or less days at hospital. The median cost of hospitalization in these patients was significantly lower than patients with more than 9 hospitalization days (P < 0.001) with the median of 95.87 (range: 19.19–856.63) and 507.30 dollars (range: 68.94–2397.54), respectively. The changes of cost deciles for patients with 9 or less days and patients with more than 9 days at hospital are presented in Fig. 3. As expected, all cost deciles for patients with 9 or less days were lower than patients with more than 9 days.

The distributions of the characteristics of the patients in two groups are shown in Table 1. Based on the findings, the number (%) of patients with age more than 60 years, comorbidity disease, abnormal breath pattern, SPO2 lower than 90%, abnormal ECG, BUN higher than 20 mg/dL, Cr higher than 1.3 mg/dL, platelets lower than 130 (×1000 µL), lymphocytes lower than 20%, abnormal neutrophil, LDH higher than 942 U/L, CPK higher than 342 IU/L, SGOT higher than 45 U/L, SGPT higher than 37 U/L, K higher than 5.1 mEq/L, BS higher than 105 mg/dL and patients with ICU experience were significantly higher in the patient with more than 9 hospitalization days compared to the patients with 9 or less hospitalization days. However, the number (%) of patients with married or single status, normal PT and normal NA were significantly lower in the patient with more than 9 hospitalization days but the differences were not statistically significant (Respectively, P = 0.078, P = 0.052, and P = 0.053). The distribution of other factors such as gender, residence and etc. were not statistically significant in two groups.

**Table 1:** The comparison of the characteristics of the COVID-19 patients with ≤ 9 and > 9 hospitalization days

Covariates	≤ 9 Days (n = 652) N (%)	> 9 Days (n = 257) N (%)	P-value
Gender			
Male	349 (53.53)	124 (48.25)	0.151
Female	303 (46.47)	133 (51.75)	
Age			
< 60 year	368 (56.44)	91 (35.41)	< 0.001
≥ 60 year	284 (43.56)	166 (64.59)	
Marriage status			
Married	563 (86.35)	210 (82.03)	0.078
Single	28 (4.29)	9 (3.52)	
Divorced and dead	61 (9.36)	37 (14.45)	
Residence			
City	562 (86.20)	217 (84.44)	0.495
Village	90 (13.80)	40 (15.56)	
Consumption of opiate			
No	629 (96.47)	248 (96.50)	0.985
Yes	23 (3.53)	9 (3.50)	
Co-morbidity			
No	382 (58.59)	100 (38.91)	< 0.001
Diabetes	39 (5.98)	21 (8.17)	
Cardiovascular	57 (8.74)	23 (8.95)	
Hypertension	98 (15.03)	50 (19.46)	
Both	76 (11.66)	63 (24.51)	
Systolic BP (mm Hg)			
> 90	643 (98.92)	255 (99.22)	0.740*
≤ 90	7 (1.08)	2 (.78)	
Breath pattern			
Normal	66 (13.1)	15 (8.5)	0.040
Takipeneh	413 (82.1)	146 (82.5)	
Dispeneh and distress	24 (4.8)	16 (9.0)	
Pulmonary lung			
Normal	509 (78.07)	199 (77.43)	0.715
Crackling (rales)	131 (20.09)	55 (21.40)	
Wheezing	12 (1.84)	3 (1.17)	
Number of breath (per minute)			
≤ 30	629 (96.77)	247 (96.48)	0.829
> 30	21 (3.23)	9 (3.52)	
Heart beat (per minute)			
≤ 125	628 (96.76)	252 (98.05)	0.294
> 125	21 (3.24)	5 (1.95)	
SPO2 (%)			
> 90	243 (37.27)	24 (9.34)	< 0.001
≤ 90	409 (62.73)	233 (90.66)	
ECG			
Normal	517 (82.9)	181 (72.7)	0.001
Abnormal	107 (17.1)	5 (27.3)	
CRP			
Negative	160 (26.14)	46 (19.01)	0.028
Positive	452 (73.86)	196 (80.99)	
ESR			
Normal	55 (9.06)	16 (6.43)	0.204
Abnormal	552 (90.94)	233 (93.57)	
BUN (mg/dL)			
5–20	513 (79.29)	168 (66.14)	< 0.001
> 20	134 (20.71)	86 (33.86)	
Cr (mg/dL)			
0.8–1.3	460 (71.32)	178 (70.08)	0.025
< 0.8	84 (13.02)	21 (8.27)	
> 1.3	101 (15.66)	55 (21.65)	
PT (Sec, n = 366)			
11–13	350 (62.28)	166 (69.46)	0.052
> 13	212 (37.72)	73 (30.54)	
PTT (Sec)			
25–35	304 (54.58)	116 (48.54)	0.118
> 35	253 (45.42)	123 (51.46)	
Platelet (×1000 µL)			
130–400	543 (84.84)	202 (78.60)	0.024
< 130	97 (15.16)	55 (21.40)	
WBC (×1000 µL)			
4.5–11	418 (65.21)	164 (63.81)	0.437
< 4.5	177 (27.61)	68 (26.46)	
> 11	46 (7.18)	25 (9.73)	
Lymphocytes (%)			
20–40	417 (63.96)	126 (49.03)	< 0.001
< 20	219 (33.59)	129 (50.19)	
> 40	16 (2.45)	2 (.78)	
Monocytes (%)			
2–8	438 (81.56)	186 (81.58)	0.802*
< 2	94 (17.50)	41 (17.98)	
> 8	5 (.93)	1 (.44)	
Neutrophils (%)			
40–60	119 (18.71)	25 (9.80)	0.001
< 40	3 (.47)	5 (1.96)	
> 60	514 (80.82)	225 (88.24)	
HCT			
Normal	453 (70.89)	170 (66.15)	0.267
Low	155 (24.26)	69 (26.85)	
High	31 (4.85)	18 (7.00)	
HB			
Normal	474 (73.83)	182 (70.82)	0.568
Low	151 (23.52)	69 (26.85)	
High	17 (2.65)	6 (2.33)	
LDH (U/L)			
≤ 942	539 (93.90)	186 (84.55)	< 0.001
> 942	35 (6.10)	34 (15.45)	
CPK (IU/L)			
≤ 342	450 (87.89)	156 (77.61)	0.001
> 342	62 (12.11)	45 (22.39)	
SGOT (U/L)			
≤ 45	447 (76.15)	152 (62.04)	< 0.001
> 45	140 (23.85)	93 (37.96)	
SGPT (U/L)			
≤ 37	444 (77.22)	169 (69.83)	0.026
> 37	131 (22.78)	73 (30.17)	
Alp (U/L)			
< 270	477 (87.52)	199 (86.90)	0.812
≥ 270	68 (12.48)	30 (13.10)	
K (mEq/L)			
3.5–5.1	36 (5.52)	18 (7.00)	0.024
< 3.5	588 (90.18)	217 (84.44)	
> 5.1	28 (4.29)	22 (8.56)	
NA (mEq/L)			
136–145	443 (69.11)	157 (62.30)	0.053
< 136	190 (29.64)	94 (37.30)	
> 145	8 (1.25)	1 (.40)	
BS (mg/dL)			
70–105	175 (37.15)	48 (22.75)	< 0.001
≥ 105	296 (62.85)	163 (77.25)	
ICU			
No	547 (84.0)	102 (39.7)	< 0.001
Yes	104 (16.0)	155 (60.3)	

The relationship of the various potential risk factors with the median hospitalization cost for patients with 9 or less and more than 9 hospitalization days is given in Table 2 using multivariable quantile regression. Based on the reported results for the patients with hospitalization ≤ 9 days in the first panel of this table, some factors had positive and significant relationship with the median of cost. The adjusted estimates presented that the median cost of hospitalization was statistically increased in patients with a history of cardiovascular disease (B = 0.28; P = 0.003), wheezing pulmonary lung (B = 0.4; P = 0.042), SPO2 lower than 90% (B = 0.2; P < 0.001), positive CRP (B = 0.2; P = 0.002), LDH higher than 942 U/L (B = 0.46; P < 0.001), NA lower than 136 mEq/L (B = 0.15; P = 0.012), lymphosite lower than 20% (B = 0.13; P = 0.013) and the patients with ICU experience (B = 0.69; P < 0.001). Although the adjusted estimate of hospitalization cost for female was more than the male, the differences was not statistically significant (P = 0.058).

**Table 2:** The association between the median of hospitalization cost and the potential risk factors using quantile regression in patients with ≤ 9 and > 9 hospitalization days

Covariate	≤ 9 Days		> 9 Days	
	Estimate (95% CI)	P-value	Estimate (95% CI)	P-value
Gender (male)				
Female	0.10 (0.0,0.20)	0.058	0.09 (− 0.13,0.31)	0.431
Age (< 60 year)				
≥ 60 year	− 0.08 (− 0.19,0.03)	0.172	− 0.27 (− 0.51,− 0.02)	0.031
Comorbidity disease (no)				
Diabetes	− 0.01 (− 0.22,0.20)	0.924	− 0.28 (− 0.66,0.10)	0.147
Cardiovascular	0.28 (0.10,0.46)	0.003	0.43 (0.0,0.86)	0.048
Hypertension	0.09 (− 0.06,0.23)	0.241	− 0.01 (− 0.32,0.3)	0.969
Multiple	− 0.04 (− 0.21,0.12)	0.619	− 0.11 (− 0.41,0.19)	0.473
Pulmonary lung (normal)				
Crackling (rales)	− 0.03 (− 0.16,0.10)	0.644	− 0.04 (− 0.3,0.22)	0.780
Wheezing	0.4 (0.02,0.78)	0.042	0.36 (− 0.47,1.20)	0.392
SPO2 (> 90)				
≤ 90	0.2 (0.09,0.31)	< 0.001	0.04 (− 0.32,0.39)	0.844
CRP (negative)				
Positive	0.2 (0.08,0.32)	0.002	0.08 (− 0.20,0.35)	0.586
PT (11–13 Sec)				
> 13	− 0.07 (− 0.17,0.04)	0.216	− 0.19 (− 0.42,0.05)	0.125
LDH (≤ 942 U/L)				
> 942	0.46 (0.25,0.67)	< 0.001	0.04 (− 0.24,0.32)	0.788
NA (136–145 mEq/L)				
< 136	0.15 (0.03,0.27)	0.012	0.09 (− 0.12,0.30)	0.391
> 145	0.37 (− 0.13,0.86)	0.144	0.52 (− 0.90,1.94)	0.469
K (3.5–5.1 mEq/L)				
< 3.5	− 0.17 (− 0.37,0.04)	0.117	− 0.16 (− 0.57,0.25)	0.434
> 5.1	0.21 (− 0.17,0.59)	0.282	− 0.07 (− 0.64,0.51)	0.821
Lymphocytes (20–40%)				
< 20	0.13 (0.01,0.24)	0.039	0.05 (− 0.18,0.28)	0.667
> 40	0.41 (− 0.12,0.94)	0.131	0.12 (− 1.38,1.61)	0.879
WBC (4.5–11 mg/dL)				
< 4.5	0.03 (− 0.09,0.15)	0.634	− 0.31 (− 0.56,− 0.05)	0.018
> 11	0.08 (− 0.12,0.28)	0.417	0.15 (− 0.27,0.57)	0.474
ICU (no)				
Yes	0.69 (0.54,0.84)	< 0.001	1.01 (0.79,1.22)	< 0.001

According to the second panel of Table 2, similar to the previous group, we observed a positive and significant association between patients history of cardiovascular disease and ICU admission to their median hospitalization cost in the patients with > 9 hospitalization days. However in the second group, these estimates were slightly higher than the first group. Moreover the adjusted estimate of patients with the age older than 60 years and WBC lower than 4.5 mg/dL had a negative and significant relationship with the median of cost (B = − 0.27, P = 0.036; B = − 0.31, P = 0.018, respectively). No significant association was observed between the median cost and PT and K in both groups.

## Discussion

This study set up to investigate the cost contributors among the COVID-19 patients in Hamadan city, the west of Iran. The results of our study revealed that there was significant difference in the median cost over hospitalization periods. In addition the median cost in each period was related to the different demographic and clinical variables.

The largest part of the direct medical cost was related to the drugs and supplies category that are not similar to another study in Fars province of Iran. They observed that hoteling was the greatest cost phenomena [2]. Difference in the cost categories could explain disagreement between our study and their results. In addition differences in the treatment regimens could be another explanation for this difference.

Quantile regressions showed that there were different variables that could explain the median costs in both hospitalization times. Regarding demographic variable of age, in the extended hospitalized patients, infected patients who were ≥ 60 years old had less median cost that could be attributed to a worsen infection status in the aged patients [13] and earlier death in them. A study in the Turkey reported similar sign; however, their result was not statistically significant [14]. Ohsfeldt et al. in the US found that by increasing in the patient age, hospitalization cost decreased that is in line to the present finding [15]. Another study in the Brazil showed that hospitalization costs decreased by increasing in age, too [16].

However, the results of a systematic review showed that being a man was related to the severe COVID-19 [13], in our study, gender did not change the median cost in both hospitalization periods. Other studies in the US and the Brazil showed that men had a higher cost compared to women [15, 16]. However, they did not divide patients based on the length of stay. Differences in the population and the treatment procedures could explain disagreement between our findings and their results. As in Miethke-Morais et al. study most of the patients were male, with at least one comorbidity and ICU admission history [16]. In Ohsfeldt et al. study too they divided patients in to groups: hospital and ICU admission [15].

It was expected that comorbidities have led to a more complicated health status in COVID-19 patients and consequently more utilization of hospital resources. Our study showed that diabetes was not related to the median of hospitalization costs in both groups that is opposite to Ohsfeldt et al. study [15]. Regarding other comorbidities, suffering from cardiovascular disease as a risk factor for the disease severity and death [13] increased the median cost in both groups that is in line to another study in Iran [8].

Patients’ lung situation was assumed to be related to their resource utilization and costs. The study’s results showed that patients with wheezing pulmonary lung compared to the cases with normal lung was only related to a higher median cost in the less hospitalized patients and by increasing in the hospitalization time this relationship was no more statistically significant. It seems by extending in the hospitalization period, the severity of disease in patients was almost homogenous. In addition, maybe by extending in the hospital stay patients need to hospital treatment were similar. As other studies showed longed hospitalization was related to more ventilator use [7, 17].

Studies showed that hypoxia is one of the common risk factors for COVID-19 severity [13]. Along to this finding, our study revealed that in patients who were hospitalized for ≤ 9 days, by decreasing in the SPO2 level the median cost increased. However, in the extended hospitalization, oxygen deficiency was no longer a cost predictor. Maybe by extending in the hospitalization period, hypoxia was more common among the long stayed patients that in turn leaded to a similar cost pattern. Ohsfeldt et al. found too that patients who needed to oxygen had a higher cost [15].

Based on our previous expectations, positive CRP increased the median cost; however, this relationship was seen only in the patients with ≤ 9 hospitalization days. Higher infection level in term of a higher inflammation level and consequently higher CRP [18] could explain more severity of disease in these patients. As Ramadan et al. reported elevated CRP was related to the disease severity [19]. However, by increasing in the hospitalization period this relationship was no more significant that could be attributed to the more prevalence of positive CRP in the prolonged infected patients.

Sodium disorders had been shown to be a predictor for COVID-19 severity. A study in the China showed both hyponatremia and hypernatremia were related to the higher disease severity, higher length of stay, and more mortality rate [20]. The results of the present study showed that NA deficiency was related to a higher hospitalization median cost, however this relationship was only significant for the less hospitalized patients.

Lower lymphocytes counts are related to the more severe disease [17, 21, 22] and a higher mortality rate in the COVID-19 patients. In shorten hospitalized patients; a lower lymphocytes level increased the median cost. This finding could be explained by higher% of patients with lower lymphocytes level in the prolonged hospitalized patients that could be related to a similar disease severity and resource utilization among them.

Regardless to the hospitalization time, patient admitted to the ICU had a higher median cost that could be related to their higher disease severity and their needs to more specialized cares. As many studies had shown ICU admission is one of the most common predictors for disease severity [7]. EKİNGEN et al. too found that hospitalization unit was a positive predictor for patients costs [14].

Other studies showed that abnormality in LDH is related to COVID-19 severity [13]. In the present study, higher LDH level was related to the increased median costs. However this relationship was true only for the less hospitalized patients and the cost of the prolonged patients did not show any statistically significant differences regarding their LDH level. Maybe failure in the respiratory function of the less hospitalized patients could explain our finding. As Poggiali et al. showed LDH may be related to the failure in the respiratory function [23].

This study was one of the first studies that evaluated driving factors of direct hospitalization costs in the COVID-19 patients in Iran. But there were some limitations that should be noted. First, we did not have data on the treatment regimens that could be related to the patients' costs. Second, the time from infection onset to the hospital admission were not available that could be related to the patients hospitalization costs. Third, variants of COVID-19 virus could be an important factor in the severity of disease and utilization pattern of patients from healthcares that were not recorded in the patients’ history.

## Conclusion

This study examined a less studied economic aspect of COVID-19 pandemic. Our results showed the importance of early treatment of the disease and prevention from the disease up to severe stages. As the length of hospital stay, which can be associated with the severity of the disease, increases, health systems become more vulnerable in terms of resource utilization, which in turn can challenge their responsiveness and readiness to meet the specialized treatment needs of individuals.

## Data Availability

The corresponding author (ETG) and another investigator (VRD) have access to the dataset. The data set analyzed during the current study is available from the corresponding author on reasonable request.
